# The influence of metacognition monitoring on L2 Chinese audiovisual reading comprehension

**DOI:** 10.3389/fpsyg.2023.1133003

**Published:** 2023-02-20

**Authors:** Yamin Wang, Jingying Hu, Zhuoma An, Chaoran Li, Yang Zhao

**Affiliations:** School of Chinese as a Second Language, Peking University, Beijing, China

**Keywords:** metacognition monitoring, measurement indicators, audiovisual reading comprehension, video difficulty, language proficiency

## Abstract

Metacognition monitoring is the ability to evaluate the cognitive process actively. L2 learners with high metacognition monitoring ability can better monitor reading processes and outcomes consciously, thus facilitating self-regulated learning and improving reading efficiency. Previous studies mostly used offline self-reports to examine the metacognition monitoring in static text reading by L2 learners. This study investigated the effects of different indicators of metacognition monitoring on L2 Chinese audiovisual comprehension by online confidence judgment and audiovisual comprehension tasks. Target measures of metacognition monitoring included absolute calibration accuracy based on video or test and relative calibration accuracy measured by Gamma or Spearman correlation coefficient. 38 intermediate-advanced Chinese learners participated in the study. Multiple regression analysis showed three main results. First, absolute calibration accuracy can significantly predict L2 Chinese audiovisual comprehension, while relative calibration accuracy has no significant effect. Second, the predictive effect of video-based absolute calibration accuracy is affected by the video difficulty, that is, the greater the video difficulty, the greater the impact on the performance of audiovisual comprehension. Third, the predictive effect of test-based absolute calibration accuracy is influenced by the language proficiency, specifically, the higher the L2 Chinese proficiency, the stronger the prediction on the performance of audiovisual comprehension. These results support a multidimensional view of metacognition monitoring by specifying how different indicators of metacognition monitoring may predict L2 Chinese audiovisual comprehension. The findings have important pedagogical implications for strategy training of metacognition monitoring and point to the necessity to take task difficulty and individual differences among learners into full consideration.

## 1. Introduction

Metacognition monitoring refers to an individual’s ability to actively assess cognitive processes, where people use appropriate and effective strategies to regulate their cognitive processes (Lin and Zabrucky, 1998). In the context of reading comprehension, metacognition monitoring is specifically concerned with the identification of difficulties and the use of strategies in the reading process, which focuses on how learners monitor their reading processes and outcomes consciously (Chen, 2009). Numerous studies have shown that metacognition monitoring has significant predictive power on the reading comprehension performance of native and L2 speakers (Garner, 1980; Maki et al., 1994; Van Gelderen et al., 2004; Taki, 2016; Silawi et al., 2020). Epstein et al. (1984) explained that learners’ self-assessment of text comprehension often does not match the actual outcomes, and cognitive illusion can affect or even hinder the whole information processing and integration to some extent. This self-assessment ability is particularly crucial in the comprehension of audiovisual multimodal texts, which involves the selection and processing of multiple information such as pictures, sounds, and texts. In addition, learners also need to integrate and evaluate the complex and dynamic information in real time to understand and interpret text meaning (Coiro, 2011; Afflerbach et al., 2015; Fox and Alexander, 2017), which poses new challenges for metacognition monitoring in multimodal text reading for second language learners.

Previous studies mainly focused on the role of metacognition monitoring on L2 reading comprehension of static texts. Van Gelderen et al. (2004) explored the metacognition monitoring strategies during L2 English reading by questionnaire surveys and showed that metacognitive knowledge significantly predicted learners’ reading comprehension performance. Taki (2016) adopted the same measure and found that metacognitive knowledge also had a significant predictive effect on Dutch L2 learners’ reading test scores. Accordingly, the above studies examined the use of explicit strategic knowledge of L2 learners mainly by offline self-reports, but Ackerman and Goldsmith (2008) argued that the basis of learning process moderation lies in continuous real-time assessment and monitoring. Obviously, it is necessary to further explore the role of metacognition monitoring on L2 reading comprehension through online test methods. In addition, it has been shown that the effect of metacognition monitoring on reading comprehension is also influenced by text difficulty and language proficiency. Kim (2014) found that text difficulty affected the metacognition monitoring strategies in English L2 online reading comprehension, and learners tended to adopt more metacognitive strategies for regulation when reading more complex texts, which had a greater impact on their reading comprehension. Furthermore, low-level L2 learners were not good at using metacognitive strategies to monitor the reading comprehension process, while high-level learners could self-assess more accurately (Han and Stevenson, 2008; Tsai et al., 2010; Míguez-Álvarez et al., 2021; Gu and Wang, 2022). It is worth mentioning that Maki et al. (2005) combined two factors of text difficulty and language proficiency. They found that English native speakers with lower language proficiency over-assessed their reading performance when reading more difficult texts, while native speakers with higher language proficiency tended to underestimate their performance. It remains to be addressed whether the predictive effect of metacognition monitoring on L2 audiovisual reading comprehension is influenced by text difficulty and language proficiency.

Related studies have also investigated the effectiveness of metacognitive abilities in Chinese L2 static text reading, mainly using think-aloud protocol (Liu, 2002), questionnaires (Wu, 2016), or interviews (Zhu and Kong, 2017). The results showed that metacognitive ability could effectively predict Chinese L2 reading comprehension performance. However, there are still some unsolved problems: (1) previous studies mainly adopted offline self-reports, and online testing methods can be conducted to explore the whole process of metacognitive monitoring; (2) metacognitive monitoring in Chinese L2 audiovisual comprehension has not yet been investigated, which may bring greater challenges to L2 learners; (3) some important factors (such as video difficulty and language proficiency) have not been examined simultaneously in previous studies. The present study aims to examine the role of metacognition monitoring on Chinese L2 learners’ audiovisual reading comprehension through online confidence judgment and audiovisual comprehension tasks and further explore the moderating effects of video difficulty and language proficiency.

## 2. Measuring metacognition monitoring

How to measure metacognitive monitoring effectively has become an essential issue in academic circles. Thiede et al. (2011) also argued that the influence of metacognition monitoring on learning outcomes cannot be emphasized enough, but it is how to be measured that is critical. Throughout previous research, the measures of metacognition monitoring in reading comprehension have consisted of two main categories: first, having learners make direct self-reports stating their use of strategic knowledge during reading (Block, 1992; Kroll and Ford, 1992; Zabrucky and Commander, 1993); and second, requiring learners to make real-time self-assessment, thus comparing the difference between learners’ self-ratings and their actual performance (Maki et al., 1994; Lin and Zabrucky, 1998; Lin et al., 2001; Sarac and Tarhan, 2009; Kasperski and Katzir, 2013). The former mainly uses offline self-reports to retrace the use of strategic knowledge during reading, while the latter focuses on the self-assessment ability during reading. Pieschl (2009) defines *calibration* as the accuracy of the learners’ perception of his or her own performance, that is, the ability to assess comprehension accurately. The researchers asked the participants to make a confidence judgment during the reading process to self-assess their reading situation, and calculate the *calibration bias*, which is the difference between the self-assessed correct rates and the actual ones (Glenberg and Epstein, 1987; Pressley and Ghatala, 1988; Zabrucky et al., 2009; Lin and Yu, 2015).

There are many different accounts of the actual measurement of metacognition monitoring in reading comprehension. It has been discussed extensively whether metacognition monitoring can effectively predict reading comprehension performance. From the perspective of the monitoring object, metacognition monitoring is divided into two types: text-based and test-based dimension. Specifically, text-based metacognition monitoring refers to the learners’ self-assessment of text comprehension after reading the text and before taking the test, i.e., pre-test confidence judgment; in contrast, test-based metacognition monitoring refers to the learners’ self-assessment of test performance after reading the text and completing the test, i.e., post-test confidence judgment (Lin and Zabrucky, 1998; Lin et al., 2001; Pieschl, 2009). Maki et al. (1994) showed that reading comprehension was significantly correlated with test-based metacognition monitoring, but not with text-based ones. Post-test confidence judgments are indeed more accurate than pre-test ones, because the post-test judgments could provide learners with more information to understand the test itself and thus better predict their actual performance (Pieschl, 2009). However, Zabrucky et al. (2009) found that both text-based and test-based metacognition monitoring were significantly associated with learners’ reading comprehension performance. In summary, the predictive effect of text-based metacognition monitoring on reading comprehension is widely divergent and needs further verification.

In addition, metacognition monitoring can be divided into two types according to the differences in statistical methods: (1) Absolute Calibration Accuracy (also known as Calibration Bias) refers to the absolute difference between the learners’ self-assessment and actual performance; the greater the absolute value of the subtraction between the two, the greater the gap between the learners’ self-assessment and actual performance; (2) Relative Calibration Accuracy (also known as Resolution) refers to the extent to which learners’ self-assessment scores reflect their actual reading comprehension scores, by calculating the correlation between the two correct rates. Higher correlation coefficients indicate greater agreement between learners’ self-assessment and actual performance (Dunlosky and Thiede, 2013). These two calculations of metacognition monitoring are statistically independent of each other, and a high absolute calibration accuracy of learners does not mean a high relative calibration accuracy and vice versa (Koriat et al., 2002; Maki et al., 2005; Griffin et al., 2009). The multidimensional view of metacognitive monitoring argues that different indicators of metacognitive monitoring have different predictive effects on reading comprehension, both in terms of monitoring objects and statistical methods, and opposes the one-dimensional view that confuses all indicators (Moore et al., 2005; Chen and Li, 2008; Hadwin and Webster, 2013). Hadwin and Webster (2013) suggested that relevant researchers should use both measures of metacognition monitoring in their actual studies to explore the relationship between the different indicators of metacognition monitoring and reading comprehension.

The present study focuses on the metacognition monitoring of L2 Chinese audiovisual reading comprehension and examines the following four measures: (1) video-based absolute accuracy, the absolute difference between learners’ self-assessment of video comprehension and their actual performance after watching the video and before taking the test; and (2) test-based absolute accuracy, the absolute difference between learners’ self-assessment of their performance on the test items and their actual performance after watching the video and completing the test; and (3) test-based relative accuracy measured by Gamma, the correlation coefficient between learners’ self-assessment of their performance on the test items and their actual performance after watching the video and completing the test; and (4) test-based relative accuracy measured by Spearman, the correlation coefficient between learners’ self-assessment of their performance on the test items and their actual performance after watching the video and completing the test. Considering the four indicators of metacognition monitoring, this study will further investigate the following two questions:

1. Do the four indicators of metacognition monitoring have a significant predictive effect on the performance of L2 Chinese audiovisual reading comprehension?
2. Is the predictive effect mediated by video difficulty and Chinese language proficiency?

## 3. Materials and methods

### 3.1. Participants

The participants were 38 undergraduate students studying Chinese at a university in Beijing (18 male, 20 female; mean age = 21.53, *SD* = 2.05). All the participants were native Korean speakers and had studied Chinese for a mean of 6.81 years (*SD* = 3.65). They were intermediate and advanced Chinese L2 learners, with an average score of 25.47 (*SD* = 3.07, ranging from 19 to 30) in the fixed-ratio cloze test (full score: 30, Feng et al., 2020). The participants had no medical history of learning disabilities, attention deficit, hearing or visual impairment. They were recruited through experimental advertisements, gave informed consent to participate in the experiment, and were paid after the experiment.

### 3.2. Measures

#### 3.2.1. Language proficiency test

In order to ensure the validity and immediacy of the test results, we did not use the participants’ acquired HSK scores as the basis for measuring their language proficiency, whereas we remeasured the participants’ Chinese language proficiency from both subjective and objective perspectives: (1) Language proficiency self-assessment questionnaire. For the subjective measures, we used the Chinese translation of the Language Experience and Proficiency Questionnaire (LEAP-Q; Marian et al., 2007). In addition to the Chinese language learning experience survey, the participants rated their Chinese language proficiency (on a 10-point scale) in four areas: listening, speaking, reading, and writing. We used the total score of the four items as the language proficiency self-ratings. (2) Fixed-ratio cloze questions. For the objective measures, we adopted the Chinese proficiency test developed by Feng et al. (2020), which required participants to complete a test of 30 fill-in-the-blanks within 15 min. If participants could not complete or fill in the blanks incorrectly, they were counted as errors (0 points), and the test had a total score of 30 points. The final score of the test is the number of correct answers (*Cronbach’s alpha* = 0.71). The Pearson correlation coefficient between the subjective and objective measures of Chinese language proficiency in this experiment was 0.602 (*p* < 0.001), indicating a strong correlation. This study followed Silawi et al. (2020) in selecting a more accurate objective measure for data analysis.

#### 3.2.2. Metacognition monitoring tasks

Participants were required to make confidence judgments before and after the test to further calculate the metacognition monitoring of the video and the test dimension. (1) Prediction confidence judgments. After watching the video and before taking the test, participants were asked, “How accurate do you think you will be when responding to comprehension questions regarding the video you have just watched?” They made judgments using a five-point scale ranging from 0 to 100% (Ackerman and Goldsmith, 2011; Silawi et al., 2020). (2) Postdiction confidence judgments. After completing each question of the video comprehension test, participants were asked, “How confident are you that you selected the correct answer?” These questions were also answered using a five-point scale from 0 to 100%.

#### 3.2.3. Video comprehension test

Participants watched three videos in Chinese on a computer screen, each followed by five multiple-choice questions, which consisted of two detail questions, two inference questions, and one topic question (Cronbach’s alpha = 0.66). The videos and test questions were adapted from *China Focus*, a set of comprehensive language textbooks designed specifically for L2 Chinese audiovisual and speaking courses. Three videos were selected for this experiment as practice video (1 min), test video 1 (3 min), and test video 2 (3 min). To control the difficulty of the videos, we revised the vocabulary and sentence patterns of the original videos and analyzed the difficulty of the revised texts with the assistance of the *Chi-Editor*, which is designed to provide CSL teachers with difficulty grading and intelligent adaptation of reading texts and contains three core modules: text rating, word annotation, and word archive (Jin et al., 2018). The results showed that the text difficulty of the practice video is the medium level (2.42), and the text length is 130 words; the text difficulty of test video 1 and video 2 are the medium level (2.01) and the high level (3.04) respectively, and have the same text length of 607 words. On this basis, we hired a professional dubbing artist (National Mandarin Proficiency Test Level 1B) to re-dub the videos and added subtitles (Song font 4, white on black background) to the three videos using the professional video editing software *Premiere*. At the end of all tests, participants were asked to answer the following question: “Which of the two previous videos did you find more difficult?” The options were “Test video 1”, “Test video 2”, and “Same difficulty”. The results showed that 89.47% (34/38) of the participants thought that test video 2 was more difficult.

The three videos used in this experiment are all character documentaries. The practice video is selected from *China Focus: Inspirational Section* (Wang, 2016a), initially titled *The Podium of Life*, which is about disabled table tennis coaches. Test video 1 is selected from *China Focus: Professional Section* (Wang, 2016b), initially titled *Beijing Cabbie*, which is about Beijing taxi drivers. Test video 2 is from *China Focus: Arts Section* (Wang, 2017), originally titled *Make the Future Better*, which is about the founder of *Meitu Xiu Xiu* software. At the end of the experiment, it was revealed through the interviews that the participants had never watched the above three videos before. They were familiar with the themes of the three videos to a similar extent.

### 3.3. Procedures

Due to the epidemic, the participants could not come to the laboratory for the experiment, so all experiments in this study were completed online. Before the formal experiment, each participant filled out a questionnaire with basic information, including age, gender, education, duration of Chinese language learning, HSK level and score, etc. They then completed two online tests of language proficiency self-assessment questionnaire and fixed-ratio cloze questions. To ensure participants’ concentration and freedom from outside interference during the experiment, they were required to conduct the experiment in a quiet space. The formal experiment was conducted on the Tencent conference platform, and their computer screens had to be shared with the experiment implementer in full screen throughout the experiment. The formal experiment consisted of two parts, that is, the practice session and the test session. Participants first watched the video online and comprehended its content. Before taking the test, they were given the prediction confidence judgment on how well they understood the video. Afterwards, they were asked to carefully complete five multiple-choice questions, each of which was presented on a single screen. Immediately after making their choice, they would assess how likely they were to get the question right and then move on to the next test question until the test was completed. With the exception of the practice session, neither the videos nor the test questions were allowed to be re-watched or redone. After viewing each video and completing the corresponding test questions, participants were given a 15 sec break. The experimental results were mainly recorded for the correctness rate of test questions and the participants’ confidence judgment scores for each video and each test question. The specific experimental procedures and materials are available at the link: https://www.wenjuan.ltd/s/3umUZzA/.

### 3.4. Data analysis

Multiple regression analysis was conducted to examine the influence of metacognition monitoring on L2 Chinese audiovisual reading comprehension using the *lm* package in the R runtime environment (R version 4.0.3; R Core Team, 2020). The dependent variable of the model was the audiovisual comprehension performance, i.e., the actual percentage of correct responses to the multiple-choice questions in the formal experimental video, and the following fixed effects were included in the model: (1) Chinese language proficiency: fixed-ratio cloze test scores; (2) video difficulty: test video 1 is an easy video and test video 2 is a more difficult video; (3) video-based absolute accuracy: the absolute value of the self-assessed correctness rate minus the actual correctness rate for each video; (4) test-based absolute accuracy: the absolute value of the self-assessed correctness rate minus the actual correctness rate for each test question; (5) test-based relative accuracy measured by Gamma: the Gamma correlation coefficient between the self-assessed and actual correctness rate of five test questions in each video; (6) test-based relative accuracy measured by Spearman: the Spearman correlation coefficient between the self-assessed and actual correctness rate of five test questions in each video. Video difficulty was a categorical variable, and the rest were continuous variables. The optimal multiple linear regression model can be fitted better if there is a certain correlation between the independent variables and the dependent variable (correlation coefficient is greater than 0.3) and the correlation between each independent variable is not too high (correlation coefficient is less than 0.7) (Pallant, 2007). Therefore, we conducted the bivariate correlation analysis and eliminated the independent variables that were not strongly correlated with the dependent variable or had multicollinearity each other before establishing the regression model.

## 4. Results

### 4.1. Preliminary analyses

Descriptive statistics and correlation analyzes are shown in Table 1. Among the four indicators measuring metacognition monitoring, there was a significant positive correlation between video-based and test-based absolute accuracy, and the two indicators of relative accuracy showed a highly positive and significant correlation. The correlation between the two relative indicators was more significant than that between the two absolute indicators. The correlations between all variables were less than 0.7. In terms of the correlation between the independent and dependent variables, there was a significant negative correlation between video-based absolute accuracy and audiovisual comprehension performance, and a borderline significant correlation between Chinese language proficiency and audiovisual comprehension performance. Univariate linear regressions were conducted on the dependent variable of audiovisual comprehension performance using each of the four indicators as the independent variable. The results showed that both two indicators of relative accuracy were not significant. Based on the data from this study, neither of the two relative indicators significantly predicted the audiovisual comprehension performance, so the model data for the absolute accuracy of metacognition monitoring were mainly reported below.

**Table 1:** Descriptive statistics and correlation matrix.

	*M*	*SD*	1	2	3	4	5	6
1. Audiovisual comprehension performance	73.16	26.64	1.00					
2. Chinese language proficiency	25.47	3.07	0.22	1.00				
3. Video-based absolute accuracy	21.84	17.41	−0.35**	−0.08	1.00			
4. Test-based absolute accuracy	20.00	17.24	−0.10	−0.02	0.37**	1.00		
5. Test-based relative accuracy measured by Gamma	0.41	0.75	−0.10	−0.16	−0.01	−0.26	1.00	
6. Test-based relative accuracy measured by Spearman	0.28	0.40	−0.01	−0.09	−0.07	−0.26	0.93***	1.00

*p < 0.05, **p < 0.01, ***p < 0.001.

### 4.2. Multiple regression analyses

The results of the multiple regression model with video-based absolute accuracy as the independent variable are shown in Table 2. The main effect of video-based absolute accuracy was marginally significant (*β* = −0.52, *SE* = 0.27, *t* = −1.94, and *p* = 0.056), indicating that larger absolute accuracy seemed to be associated with poorer audiovisual comprehension performance. The effect of video difficulty was significant (*β* = −97.85, *SE* = 37.37, *t* = −2.62, and *p* = 0.011), with the more difficult the video, the worse the comprehension performance. Importantly, we found a significant interaction effect between video-based absolute accuracy and video difficulty (*β* = −0.97, *SE* = 0.31, *t* = −3.17, and *p* = 0.002), with the follow-up contrasts demonstrating the greater the video difficulty, the greater the effect of video-based absolute accuracy on audiovisual comprehension performance. The effect of Chinese language proficiency was not significant (*β* = −0.17, *SE* = 1.00, *t* = −0.17, and *p* = 0.863), but the interaction effect of video difficulty and Chinese language proficiency was significant (*β* = 3.42, *SE* = 1.43, *t* = 2.40, and *p* = 0.019); specifically, the effect of Chinese language proficiency on audiovisual comprehension performance was enhanced with the increase of video difficulty.

**Table 2:** Multiple regression of audiovisual comprehension performance based on video-based absolute accuracy.

Variables	*β*	*SE*	*t*	*p*
Intercept	84.67^**^	25.72	3.29	0.002
Video-based absolute accuracy	−0.52	0.27	−1.94	0.056
Video difficulty	−97.85^*^	37.37	−2.62	0.011
Chinese language proficiency	−0.17	1.00	−0.17	0.863
Video-based absolute accuracy: video difficulty	−0.97^**^	0.31	−3.17	0.002
Video difficulty: Chinese language proficiency	3.42^*^	1.43	2.40	0.019

*R*^2^ = 0.5367, *F*(5, 70) = 16.21, *p < 0.05, **p < 0.01.

A similar set of multiple regression analyzes were also conducted with test-based absolute accuracy as the independent variable (see Table 3). Test-based absolute accuracy contributed significantly to audiovisual comprehension performance (*β* = −2.98, *SE* = 1.17, *t* = −2.55, and *p* = 0.013), with the higher the deviation value of the self-assessment, the worse the audiovisual comprehension performance. The main effect of video difficulty was also significant (*β* = −34.20, *SE* = 7.62, *t* = −4.49, and *p* < 0.001), which indicated the more difficult the video, the worse the audiovisual comprehension performance. Chinese language proficiency was not a significant predictor in the model (*β* = −0.87, *SE* = 1.18, *t* = −0.73, and *p* = 0.467), while the interaction effect of test-based absolute accuracy and Chinese language proficiency was significant (*β* = 0.14, *SE* = 0.04, *t* = 3.10, and *p* = 0.003). Further comparisons revealed that the effect of test-based absolute accuracy on audiovisual comprehension performance increased with the improvement of Chinese language proficiency. The model yielded no significant interaction between test-based absolute accuracy and video difficulty (*β* = −0.29, *SE* = 0.38, *t* = −0.77, and *p* = 0.442).

**Table 3:** Multiple regression of audiovisual comprehension performance based on test-based absolute accuracy.

Variables	*β*	*SE*	*t*	*p*
Intercept	106.43**	31.28	3.40	0.001
Test-based absolute accuracy	−2.98*	1.17	−2.55	0.013
Video difficulty	−34.20***	7.62	−4.49	<0.001
Chinese language proficiency	−0.87	1.18	−0.73	0.467
Test-based absolute accuracy: video difficulty	−0.29	0.38	−0.77	0.442
Test-based absolute accuracy: Chinese language proficiency	0.14**	0.04	3.10	0.003

*R*^2^ = 0.5264, *F*(5, 70) = 15.56, *p < 0.05, **p < 0.01, ***p < 0.001.

## 5. Discussion

### 5.1. The predictive effect of metacognition monitoring on L2 Chinese audiovisual reading comprehension

The Self-regulation Learning Theory suggests that learners need to monitor their learning in real time to see if they reach their predetermined goals (Zimmerman and Schunk, 2001), emphasizing the role of metacognition monitoring in individual learning. Our findings showed that metacognition monitoring effectively predicts L2 Chinese audiovisual reading comprehension, which are consistent with those of Chinese L2 static text reading studies (Liu, 2002; Zhu and Kong, 2017). In this study, intermediate and advanced Chinese L2 learners could make appropriate self-assessment of audiovisual reading comprehension. Specifically, the test-based absolute accuracy is a better predictor of L2 Chinese audiovisual comprehension performance than the video-based absolute accuracy, consistent with studies of native speakers (Lin et al., 2001; Pieschl, 2009; Zabrucky et al., 2009). Both two absolute indicators derived by confidence judgment task represent the absolute difference between self-assessment and actual performance. Still, their connotations reflect different psychological features of metacognition monitoring, where the temporal dimension of making confidence judgments appears to be particularly important. In the first confidence judgment, participants need to estimate the expected comprehension performance before being exposed to the test. In contrast, in the second confidence judgment, they can integrate both text and test information to assess their actual performance and obviously can obtain more additional clues to help them make more accurate judgments. In addition, Sarac and Tarhan (2009) showed that English L2 learners made more accurate self-assessments based on their test performance but were unable to accurately assess their own comprehension of the text, which the authors attributed to a lack of discourse knowledge. Although the participants in this study were all intermediate to advanced learners who may have been familiar with most of the Chinese words and grammar in the videos, they had difficulty in following up on the video story in a specific organizational development way. Moreover, new information and new ideas emerged in real time. Therefore, it was difficult to make accurate judgments based solely on the videos.

The present study did not find significant predictive effects of the two relative indicators of metacognition monitoring on L2 audiovisual reading comprehension, which may indicate that the practical predictability of the relative indicators is less stable (Ackerman and Goldsmith, 2011). Masson and Rotello (2009) also found that Gamma coefficients computed from simulated sample data would reflect variation due to item bias rather than actual accuracy. These artifactual effects tend to be exaggerated in unequal variance distributions. In the case of this study, it may also be due to two reasons: on the one hand, the Gamma and Spearman coefficient are both calculated based on fixed-order variables, and the existence of missing values in the actual calculation process resulted in the relative indicators not being able to reflect the correlation between self-assessment and actual performance (Wiley et al., 2016); on the other hand, the relatively small number of test questions, with only five questions per video, may affect the stability and sensitivity of these coefficients (Spellman et al., 2008). Some researchers have also argued that we must be cautious in using relative indicators for metacognition monitoring in practical applications because their practical predictability may be affected by factors such as sample distribution, missing calculations, and the number of items (Masson and Rotello, 2009; Ackerman and Goldsmith, 2011).

It is worth mentioning that the four indicators of metacognition monitoring were examined comprehensively in this study, and there were differences in the predictive effects on audiovisual comprehension performance. Explicitly speaking, test-based absolute accuracy could significantly be predicted audiovisual comprehension performance and video-based absolute accuracy had weaker predictive power, while the two relative indicators did not have significant predictive effects. The above results provide support for the multidimensional view of metacognition monitoring (Moore et al., 2005; Chen and Li, 2008; Hadwin and Webster, 2013), demonstrating that the validity and stability of metacognition monitoring indicators vary, whether they are video-based or test-based, absolute or relative. Their practical predictability still need to be further examined in the context of specific experimental scenarios and instruments. Therefore, a one-dimensional view that simply handles different indicators without being distinguished should be opposed.

### 5.2. Real-time measures of metacognition monitoring and L2 audiovisual reading comprehension

Previous studies have focused on the role of metacognition monitoring in static text comprehension. In contrast, this study examined metacognition monitoring in L2 audiovisual reading comprehension using real-time measures, demonstrating the effectiveness of metacognition monitoring for intermediate and advanced Chinese L2 learners. Ackerman and Goldsmith (2008) argue that the basis of regulating the learning process lies in continuous real-time assessment. Online audiovisual reading comprehension shown in our experiments is a continuous, real-time dynamic learning process. Metacognition monitoring affects the whole process of cognitive activity, and the changed cognitive processing state will in turn influence metacognition monitoring. Our results showed that two of all four metacognition monitoring indicators measured by the real-time confidence judgments were good predictors of Chinese L2 audiovisual comprehension performance, demonstrating the reliability and validity of the online measure. It also proves that intermediate and advanced Chinese L2 learners can make accurate self-assessments especially continuous based on test performance during the selection and processing of multimodal information such as pictures, sounds, and words.

In real-life contexts, self-regulated learning by native or L2 speakers is online in real time, which requires them to monitor and regulate their learning process instantaneously. Metacognition monitoring reflected in this process not only motivates learners to adopt effective strategies but also enhances their self-efficacy, thus improving the effectiveness of online learning (Chen and Wen, 2010). Ackerman and Goldsmith (2011) also found that the difference between screen reading and paper reading is usually not cognitive but mainly reflected in metacognition, that is, learners were more inaccurate in predicting online reading performance and consumed more time in regulating learning time. The present study highlights the importance of metacognition monitoring for online audiovisual reading comprehension.

### 5.3. Moderating effects of video difficulty and L2 proficiency

The Cognitive Effort Hypothesis holds that learners’ self-assessment accuracy of text comprehension is affected by text difficulty. To be specific, self-assessment of text comprehension is less accurate if the text being read is easy for the learner since they do not pay excessive attention to the details of easy texts (Maki et al., 1990). The present study found that the predictive effect of video-based absolute accuracy on Chinese L2 audiovisual comprehension was affected by video difficulty, in line with the Cognitive Effort Hypothesis. With the increase of video difficulty, the accuracy of Chinese learners’ self-assessment of video comprehension is better, and the role of metacognition monitoring in predicting audiovisual comprehension is greater. Kim, (2014) also found that English L2 learners tended to use more metacognitive reading strategies when reading difficult texts, which had a greater impact on their reading comprehension. In addition, the participants selected for the experiment are intermediate to advanced Chinese L2 learners. When learners read the easy video (video 1), they were not inclined to consume too many cognitive resources in the control of video details or adopt more metacognitive reading strategies, but could get good audiovisual comprehension scores in the end. Further analysis showed that 20 participants (38 in total) achieved an actual correct rate of 100% on the video 1 test, with an overall average correct rate of 88.95%. To some extent, there was a ceiling effect, i.e., intermediate and advanced learners generally understood the lower difficulty videos better, so the effect of metacognition monitoring on audiovisual comprehension may be weakened.

The present study also found that the predictive effect of test-based absolute accuracy on L2 Chinese audiovisual comprehension was also moderated by Chinese language proficiency, i.e., the stronger the predictive effect of metacognition monitoring on audiovisual comprehension as learners’ Chinese language proficiency increased. Tsai et al. (2010) found that language proficiency had a greater impact on English L2 speakers’ use of reading metacognitive strategies compared to native speakers, and that highly proficient L2 speakers were better able to use more metacognitive strategies to monitor their reading comprehension process, which was conducive to improving their text comprehension and thus achieving higher reading scores. Míguez-Álvarez et al. (2021) also found that the monitoring accuracy of Spanish L2 speakers is influenced by their language level, that is, learners with lower L2 proficiency tended to overestimate their reading performance, while higher-level L2 learners are better able to monitor the reading process and make more accurate self-assessments. In addition, the absolute accuracy of metacognition monitoring was significantly higher in high-level learners than in low-level learners (Gu and Wang, 2022), because high-level learners were more likely to pay attention to and identify important information in reading materials and also invested more time and cognitive resources.

The above results suggest that the predictive effect of metacognition monitoring on Chinese L2 audiovisual comprehension is complex. The prediction of video-based absolute accuracy was affected by the video difficulty (the more difficult the video, the more significant the prediction effect). In contrast, the prediction of test-based absolute accuracy was moderated by L2 proficiency (the higher Chinese L2 proficiency, the more significant the prediction effect). Our findings also provide evidential support for a multidimensional view of metacognition monitoring (Moore et al., 2005; Chen and Li, 2008; Hadwin and Webster, 2013). As stated earlier, some differences exist in L2 learners’ metacognition monitoring on the two dimensions of video comprehension and test performance, both in terms of self-assessment time and information resource capacity. Thus, their predictive effects are moderated by different variables.

## 6. Conclusion and implications

The current study systematically examined four indicators of metacognition monitoring in L2 Chinese audiovisual comprehension from the perspective of monitoring objects and statistical methods by online confidence judgment tasks. Furthermore, we explored the predictive effects of different indicators on L2 audiovisual comprehension performance. The main results showed that the absolute accuracy of metacognition monitoring had a significant predictive effect on L2 Chinese audiovisual comprehension, while the effect of relative accuracy was not significant. Specifically, the predictive effect of video-based absolute accuracy was influenced by the video difficulty (the more difficult the video, the more significant the prediction effect). In contrast, test-based absolute accuracy was moderated by L2 proficiency (the higher Chinese L2 proficiency, the more significant the prediction effect). Our findings support a multidimensional view of metacognition monitoring and oppose a one-dimensional view that simply makes no distinction among all four indicators.

Pedagogically, the empirical findings in this study not only emphasize the importance of metacognition monitoring in L2 audiovisual comprehension, but also provide some implications for teaching Chinese as a second language. Firstly, both Chinese teachers and learners should recognize that reading comprehension is not only a complex cognitive process but also a complex metacognitive process, and learners with higher metacognition monitoring ability can effectively regulate their own cognitive processes in real time and thus make more accurate self-assessments. Therefore, instructional interventions aimed at metacognition monitoring are essential. Teachers should provide learners with a variety of strategies needed to conduct more accurate self-assessment, such as planning and prediction of text content, assessment of the characteristics of text genre, assessment of a text content, construction and modification of a mental model, supervision of comprehension and text understanding, revision, and self-correction (Míguez-Álvarez et al., 2021). Secondly, this study also found that the predictive effect of metacognition monitoring on L2 audiovisual comprehension is complex and is affected by the video difficulty and language proficiency, which reveals that we need to appropriately adjust the difficulty of the video task or text according to L2 learners’ language proficiency when training metacognition monitoring strategies, so as to motivate learners to mobilize their own initiative, monitor and regulate audiovisual comprehension in real time within a specific range of cognitive effort.

## Data availability statement

The original contributions presented in the study are included in the article/supplementary material, further inquiries can be directed to the corresponding author.

## Ethics statement

The studies involving human participants were reviewed and approved by Academic Committee of School of Chinese as a Second Language, Peking University. The patients/participants provided their written informed consent to participate in this study.

## Author contributions

YZ and YW designed the study and contributed to the final manuscript writing. YW, JH, ZA, and CL performed the study and collected the data. JH and YW analyzed the data. YW, JH, ZA, and CL prepared the draft. All authors contributed to the article and approved the submitted version.

## Funding

This study was funded by National Social Sciences Project “Application of Chinese Proficiency Grading Standards and Strategies of Textbook Research and Development (21STA032)”.

## Conflict of interest

The authors declare that the research was conducted in the absence of any commercial or financial relationships that could be construed as a potential conflict of interest.

## Publisher’s note

All claims expressed in this article are solely those of the authors and do not necessarily represent those of their affiliated organizations, or those of the publisher, the editors and the reviewers. Any product that may be evaluated in this article, or claim that may be made by its manufacturer, is not guaranteed or endorsed by the publisher.
